# Structural Characterisation of the Nuclear Import Receptor Importin Alpha in Complex with the Bipartite NLS of Prp20

**DOI:** 10.1371/journal.pone.0082038

**Published:** 2013-12-10

**Authors:** Noelia Roman, Mary Christie, Crystall M. D. Swarbrick, Bostjan Kobe, Jade K. Forwood

**Affiliations:** 1 School of Biomedical Sciences, Charles Sturt University, Wagga Wagga, New South Wales, Australia; 2 School of Chemistry and Molecular Biosciences, Institute for Molecular Bioscience and Australian Infectious Diseases Research Centre, University of Queensland, Brisbane, Queensland, Australia; Institute of Enzymology of the Hungarian Academy of Science, Hungary

## Abstract

The translocation of macromolecules into the nucleus is a fundamental eukaryotic process, regulating gene expression, cell division and differentiation, but which is impaired in a range of significant diseases including cancer and viral infection. The import of proteins into the nucleus is generally initiated by a specific, high affinity interaction between nuclear localisation signals (NLSs) and nuclear import receptors in the cytoplasm, and terminated through the disassembly of these complexes in the nucleus. For classical NLSs (cNLSs), this import is mediated by the importin-α (IMPα) adaptor protein, which in turn binds to IMPβ to mediate translocation of nuclear cargo across the nuclear envelope. The interaction and disassembly of import receptor:cargo complexes is reliant on the differential localisation of nucleotide bound Ran across the envelope, maintained in its low affinity, GDP-bound form in the cytoplasm, and its high affinity, GTP-bound form in the nucleus. This in turn is maintained by the differential localisation of Ran regulating proteins, with RanGAP in the cytoplasm maintaining Ran in its GDP-bound form, and RanGEF (Prp20 in yeast) in the nucleus maintaining Ran in its GTP-bound form. Here, we describe the 2.1 Å resolution x-ray crystal structure of IMPα in complex with the NLS of Prp20. We observe 1,091 Å^2^ of buried surface area mediated by an extensive array of contacts involving residues on armadillo repeats 2-7, utilising both the major and minor NLS binding sites of IMPα to contact bipartite NLS clusters ^17^RAKKMSK^23^ and ^3^KR^4^, respectively. One notable feature of the major site is the insertion of Prp20NLS Ala^18^ between the P0 and P1 NLS sites, noted in only a few classical bipartite NLSs. This study provides a detailed account of the binding mechanism enabling Prp20 interaction with the nuclear import receptor, and additional new information for the interaction between IMPα and cargo.

## Introduction

A distinguishing feature of all eukaryotic cells is the containment of their genetic material within a stable and segregated nuclear organelle. This in turn requires the active, bidirectional transport of proteins and RNA across the nuclear envelope, a central process to a range of important cellular events including DNA replication, cell differentiation, and diseases including cancer and viral replication. In the classical nucleocytoplasmic transport pathway, the nuclear import receptor importin-α (IMPα) recognises a nuclear localisation signal (NLS) displayed on a cargo protein, and this dimer, through interaction with importin-β (IMPβ), is docked and translocated across the nuclear pore complex through interaction with nucleoporins [1]–[3]. Once in the nucleus, the IMPα:IMPβ:cargo complex is dissociated by RanGTP, and the IMPs are recycled back to the cytoplasm for a further round of import [4], [5].

The initial interaction of the nuclear import pathway between IMPα and the nuclear import cargo has been studied by a range of structural and functional approaches. IMPα, a large (529 residue) highly conserved macromolecule, is composed of 2 structural domains, a short basic 10 kDa N-terminal domain that binds importin-β (IBB domain), and a 50 kDa armadillo (ARM)-repeat NLS binding domain that recognises and binds NLSs of various cargo proteins [6], [7]. Interaction with the NLS generally occurs at the concave face of the ARM domains, at locations that are typically driven by the type of NLS; monopartite NLSs (composed primarily of a single cluster of positively charged residues) bind at the major NLS-binding site, and bipartitite NLSs, comprised of two positively charged separated by a 10–12 residue linker, bind by spanning both the major and minor sites of IMPα [7]–[9]. The usual nomenclature for describing the interactions between IMPα and NLSs [10], [11] designates residues binding in the minor site as P1′, P2′ etc., and residues binding the major site as P1, P2 etc. The consensus sequences correspond to K[K/R]X[K/R] (corresponding to positions P2–P5; [K/R] represents Lys or Arg, and X represents any amino acid) for monopartite and [K/R][K/R]X_10–12_[K/R]_3/5_ (corresponding to positions P1′–P2′ for the N-terminal basic cluster) cNLSs) for bipartite NLSs [6].

The directionality of the nuclear transport process is governed by the differential localisation of Ran across the nuclear envelope (reviewed in [12]–[14]). Specifically, the nucleotide bound state of Ran results in conformational changes in two surface loops of the protein, termed switch I and switch II, which in turn mediates its ability to dissociate importin:cargo complexes in the GTP-bound form [5], [15]. This is achieved through the asymmetric distribution of Ran regulatory proteins across the nuclear envelope, which modulate the nucleotide bound state of Ran; Ran guanidine exchange factor (RCC1/Prp20), predominately localised to the nucleus, maintains Ran in a GTP bound form that binds IMPs with high affinity and dissociates the complex upon nuclear entry; whilst RanGTPase activating protein (RanGAP), cytoplasmically located, maintains Ran in a GDP bound conformation. Thus, nuclear localisation of Prp20 plays a vital role in establishing the directionality of the nuclear localisation process.

Previous studies have elucidated the region within Prp20 responsible for its nuclear localisation [16]–[18], however the detailed mechanism of its import remained to be fully determined. The NLS region is contained within the N-terminal 25 residues, and interacts with IMPα. Here, using x-ray crystallography, we elucidate the binding interface between IMPα and the NLS region of Prp20, and provide a structural comparison to other known IMPα:NLS cargo interaction interfaces.

## Materials and Methods

### Expression and Purification

Mouse IMPαΔIBB (residues 70–529) was overexpressed using thio-β-D-galactose (IPTG) method as outlined in [19]. The sample was purified using a nickel-nitrilotriacetic acid (Ni-NTA) column (GE Healthcare), where cells were lysed by freeze-thawing in the presence of 20 mg of lysozyme, and cleared bacterial cellular extract injected onto a 5 mL HisTrap HP column (GE Healthcare) in His Buffer A (50 mM phosphate buffer, 300 mM NaCl, 20 mM imidazole, pH 8; AKTApurifier FPLC (GE Healthcare)), washed, and eluted with His Buffer B (50 mM phosphate buffer, 300 mM NaCl, 500 mM imidazole, pH 8). Peak fractions were pooled and loaded onto a HiLoad 26/60 Superdex 200 column (GE Healthcare) containing 20 mM Tris pH 7.8, 125 mM NaCl, for size exclusion chromatography, where peak fractions were collected and added to a GST column loaded with GST-tagged Prp20 (*S. cerevisiae* RanGEF) NLS (residues 3–23, KRTVATNGDASGAHRAKKMSK^23^). Prp20 NLS was overexpressed as a GST-fusion protein using the autoinduction method as previously described in [6]. GST:Prp20 NLS was injected and immobilised on a GSTrap FF column (GE Healthcare), washed extensively in binding buffer containing 50 mM Tris pH 7.8, 125 mM NaCl. Purified IMPαΔIBB was then passed over the column containing GST:Prp20 NLS, washed, and eluted in binding buffer containing 10 mM glutathione. The GST-tag was removed by overnight treatment of thrombin at 4°C, and the complex purified by a further round of size exclusion chromatography. The complex, in 20 mM Tris pH 7.8, and 125 mM NaCl, was then concentrated to 20 mg/mL (Amicon, MWCO 10 kDa, Millipore), aliquoted, and flash-frozen in liquid nitrogen and stored at −80°C.

### Crystallization and Data Collection

Single rod shaped crystals, measuring 200×50×50 µm, were obtained in 500 mM sodium citrate pH 6–8, 10 mM DTT after 2 d, harvested with a cryoprotectant composed of 80% mother liquid and 20% glycerol, and flash frozen with liquid nitrogen. Diffraction data was collected at the Australian Synchrotron (MX2) Beamline using BLU-ICE software [20]. 180° of diffraction data (0.5° oscillations) were integrated, scaled, and converted to structure factors using MosFlm [21], Scala [22] and Truncate [23].

### Structure Determination and Refinement

Diffraction images were integrated and scaled to 2.1 Å resolution in iMOSFLM, with an Rmerge of 6.7% (data statistics are summarized in Table 1). IMPα residues 72–497 from the nucleosplasmin NLS complex structure (PDB ID 3UL1) [6] were used as the search model for molecular replacement to generate phases and an initial electron density map, with the test set reflections transferred from the search model dataset. Both rigid body and restrained refinement were performed using Refmac. Prp20 backbone was built manually through iterative cycles of COOT and REFMAC [24], [25].

**Table 1:** Crystallographic data.

**Data collection**	
Space group	*P* 2_1_ 2_1_ 2_1_
Unit cell dimensions (Å)	*a* = 78.91, *b* = 89.92, *c* = 99.70
Resolution range (Å)	36.37–2.10 (2.16–2.10)^a^
Total reflections	294,256 (24,607)^a^
Unique observations	53,834 (3,413)^a^
Completeness (%)	100 (100)^a^
Multiplicity	7.0 (7.2)^a^
R_merge_ (%)^b^	0.07 (0.30)^a^
Average I/σ (I)	16.7 (6.0)^a^
Mosaicity	0.6
**Refinement**	
R_cryst_/R_free_ (%)^c^	16.7 (18.9)/20.1(20.4)
Bond length RMSD (Å)	0.022
Bond angle RMSD (°)	2.09
Average B factor (Å^2^)	34.80
Ramachandran plot (%)^d^	
Favoured	99
Outliers^e^	0.23

^a^ Numbers in parenthesis are for the highest resolution shell.

_merge_ = ∑_hkl_(∑_i_(|I _hkl_,_i_−<I _hkl_ >|))/∑_hkl_,_i_ <I _hkl_>, where I _hkl_,_i_ is the intensity of an individual measurement of the reflection with Miller indices h, k and l, and <I_hkl_> is the mean intensity of that reflection.^b^ R

_cryst_ = ∑_hkl_(||Fobs_hkl_|−|Fcalc_hkl_||)/|Fobs_hkl_|, where |Fobs_hkl_| and |Fcalc_hkl_| are the observed and calculated structure factor amplitudes. R_free_ is equivalent to R_cryst_ but calculated with reflections (5%) omitted from the refinement process.^c^ R

^d^ Calculated with the program PROCHECK

α structures^e^ Asn239 is Ramachandran outlier in all IMP

## Results and Discussion

For Prp20 to maintain Ran in its nuclear, GTP bound state, it must first be translocated to the nucleus. The region within Prp20 responsible for directing this localisation has been clearly defined [16], and shown to reside within residues 1–25. Using amino acid substitutions, the NLS was shown to be bipartite, consisting of residues KR^4^, a 12-residue spacer, and RAKKMSK^23^. The C-terminal cluster does not conform to the conventional NLS consensus. To elucidate the structural basis for the interaction between the nuclear import receptor IMPα and the NLS region of the nucleotide exchange factor Prp20, the domains previously demonstrated to mediate this interaction [16] were recombinantly expressed, purified to homogeneity, and isolated as an equimolar complex. Recombinant IMPαΔIBB (IMPα lacking the N-terminal IBB domain, thus preventing autoinhibition of the NLS binding site), consisting of 10 consecutive ARM repeat domains, was first purified by affinity and size exclusion chromatography, then applied to a column loaded with purified GST-tagged Prp20 NLS. Excess IMPαΔIBB^70–529^ was removed through extensive column washing, and subsequent elution, affinity tag-cleavage, and size exclusion chromatography enabled efficient separation of IMPαΔIBB:Prp20 NLS complex (kDa of >50 kDa) from excess Prp20 (<5 kDa), thus isolating a homogenous complex of equimolar concentrations.

**Figure 1:**
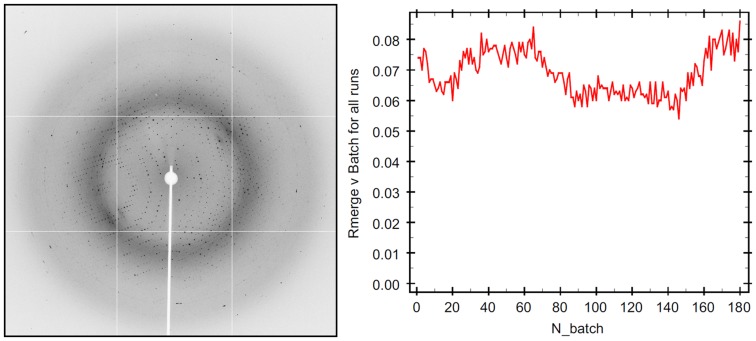
Diffraction image (left) and Rmerge statistics across batches (right), demonstrating crystal resistance to radiation damage during data collection.

**Figure 2:**
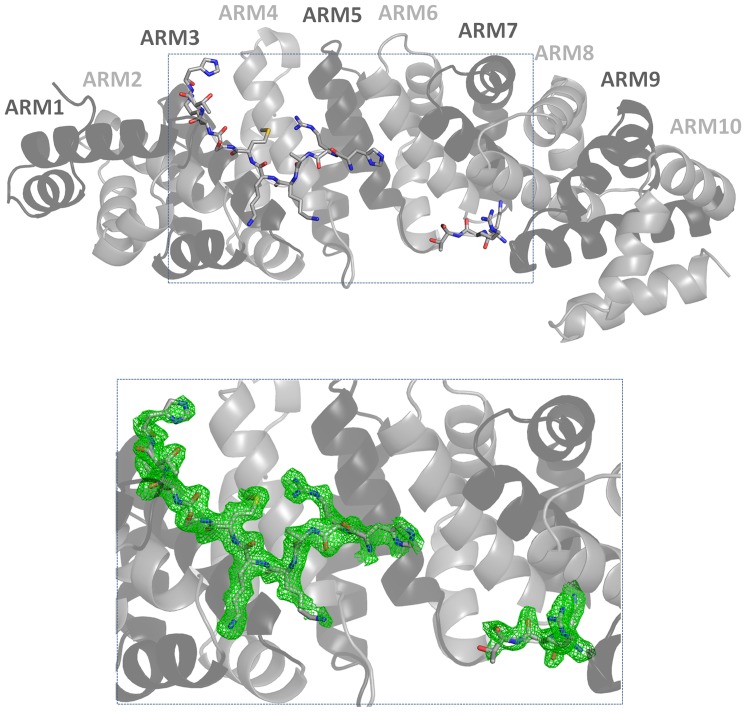
Cartoon overview of the IMPα (in ribbon):Prp20 (stick model) complex structure (top), superimposed on the Fo-Fc annealed omit map (green; calculated using Phenix [30], contoured at 2.0 σ). Figures were produced using PyMOL (DeLano Scientific LLC).

Large, strongly diffracting crystals were grown in a citrate-containing solution, based on conditions as described in the Materials and Methods section. Using synchrotron radiation, crystals diffracted to 2.1 Å resolution and were resistant to radiation damage, permitting 180° of data to be collected from a single crystal without deterioration in diffraction quality (Fig. 1). Following rigid body refinement and simulated annealing, analysis of both 2F_o_–F_c_ and simulated annealed omit maps revealed clear density in the major and minor NLS-binding sites of IMPα, enabling residues of the Prp20 NLS to be built (Fig. 2).

**Figure 3:**
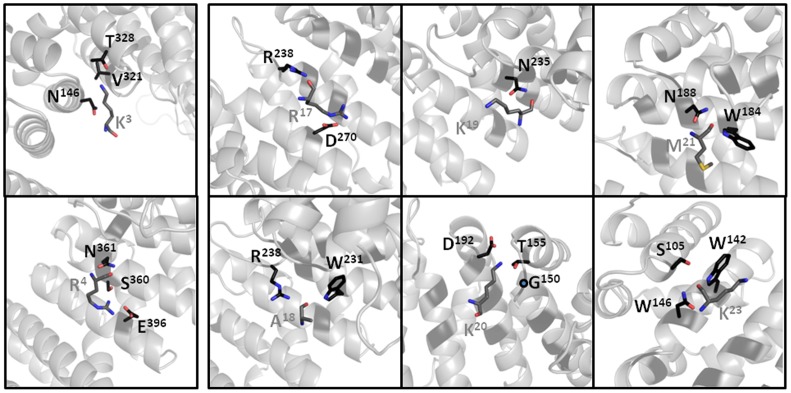
Structure of the complex between Prp20 NLS (grey sticks) and IMPα (grey cartoon backbone and black sticks), highlighting interactions at specific positions. The first two panels highlight the interactions at the minor site (NLS residues K^3^ and R^4^), and the remaining binding sites highlight the interactions at the major site (NLS residues RAKKMSK^23^). Figures were produced using PyMOL (DeLano Scientific LLC).

A final structural model, comprised of IMPα residues 72 to 497, bound to Prp20 NLS residues KR^4^ and RAKKMSK^23^, and 126 water molecules, with good stereochemistry has been deposited to the PDB (Table 1). Residues 6–15 of Prp20 could not be discerned from the electron density, a common observation for bipartite NLSs with long linker regions [6] and these residues were omitted from the final model. The 425 residues comprising IMPαΔIBB are structured into 10 ARM repeats, with an overall arrangement similar to that of available IMPα structures (e.g. RMSD for the equivalent Cα residues from the structures with PDB IDs EJY, 1EJL, 1PJM are 0.28, 0.29, and 0.30 Å, respectively). The interaction between IMPα and the Prp20 NLS is made through an extensive array of contacts involving residues contained with ARM repeats 2 through 7, utilising both the major and minor NLS binding sites of IMPα to contact ^Prp20NLS^RAKKMSK^23^ and the canonical ^Prp20NLS^KR^4^ motif, respectively, and exhibiting a total of 1,091 Å^2^ buried surface area. One notable feature of the major site is the insertion of ^Prp20NLS^Ala^18^ between the P0 and P1 NLS positions, noted in only a few classical bipartite NLSs. This results in hydrogen-bonding interactions between the ^Prp20NLS^Ala^18^, and the side chains of Trp^231^ and Arg^238^ of IMPαΔIBB (Fig 3). The interaction at the P0-binding site is mediated by a salt bridge between the guanidinium side chain of ^Prp20NLS^Arg^17^ and the carboxylate side chain of Asp^270^, as well as hydrogen bonding between the main chain of ^Prp20NLS^Arg^17^ and the side chain of Arg^238^. At the P1-binding site, the main chain of ^Prp20NLS^Lys^19^ forms hydrogen bonds with the side chain of Asn^235^ [ND2] (Table 2). The prominent P2-binding site displays multiple interactions involving a salt bridge between the ammonium side chain of ^Prp20^Lys^20^ and the carboxyl side chain of Asp^192^, as well as hydrogen bonding between side chain of ^Prp20NLS^Lys^20^ and the oxygen of the side chain of Thr^155^ and the main chain of Gly^150^. At the P3-binding site, hydrogen bonds and hydrophobic interactions are observed between the main chain of ^Prp20NLS^Met^21^ and side chains of Trp^184^ and Asn^188^. At the P5-binding pocket, the main chain of ^Prp20NLS^Lys^23^ interacts with the side chains of Ser^105^, Trp^142^, and Asn^146^, while ^Prp20NLS^Lys^23^ main chain N is hydrogen-bonded with the side chain of Asn^146^.

**Table 2:** NLS binding to the major and minor sites of IMPα.

NLS	Minor site				Linker*	Major site					PDB ID
	P1′	P2′	P3′	P4′		P1	P2	P3	P4	P5	
Prp20	K	R	T	*V*	*ATNGDASGA*HRA	K	K	M	S	K	This study
Bimax1	K	R	P	L	EWDEDEEPP	R	K	R	K	R	3UKW
Rb	K	R	S	A	EGSNPPKP	L	K	K	L	R	1PJM
NpI	K	R	P	A	ATKKAGQ	A	K	K	K	K	1EE5
yCBP80	K	R	R	G	D*FDEDENYRDFRPR*M	P	K	R	Q	R	3UKY
PB2	K	R	D	S	*SILTDS*QTA	T	K	R	I	R	2JDQ

[6] 1PJM [9], 1EE5 [28], 3UKY [6], 2JDQ [29]. Italics indicate that the sequence could not be discerned from the electron density and was omitted from the model. References for PDB ID 3UKW

The minor site involves hydrogen-bond interactions between the side chain of ^Prp20NLS^Lys^3^ with the side chain of Thr^328^, and main chain of Val^321^ and Asn^361^, whilst the P2′-binding site shows multiple interactions involving a salt bridge between the guanidinium of ^Prp20NLS^Arg^4^ and the carboxylate of Glu^396^; and hydrogen bonding between the ^Prp20NLS^Arg^4^ side chain and the main chain of Ser^360^, and the main chain of ^Prp20NLS^Arg^4^ with the side chain of Asn^361^.

The interface observed between IMPα and the Prp20 NLS side chain and main chain residues both consolidate known binding site information of IMPα, as well as provide additional new interactions previously not described [6], [9]–[11], [26], [27]. The P0-binding site, generally comprised of Asn^235^, Arg^238^, and involving hydrophobic interactions, in our structure involves the interaction with Arg^238^, however the interaction between Asn^235^ is disrupted, and instead replaced by a strong salt interaction between Arg^17^ and Asp^270^. Consistent with our structural observations, mutation of ^Prp20NLS^Arg^17^ to Thr resulted in reduced binding to IMPα [16]. The P1-binding pocket generally accommodates a long positively charged NLS side chain, despite the fact that the interaction generally involves only nonspecific interactions with the main chain of the NLS. Indeed, our structure is consistent with this general observation, with the ^Prp20NLS^Lys^19^ only interacting through its main chain with the side chain of Asn^235^. Analysis of IMPα bound to NLSs has previously revealed P2 as the most critical position in the NLS. The binding pocket is predominantly comprised of Gly^150^, Thr^155^ and Asp^192^ on IMPα, and the pocket appears best suited for binding a lysine residue; these structural observations have been confirmed through site-directed mutagenesis studies, where mutagenesis of the K to A at the P2 position not only abolished nuclear localisation of the protein, but also reduced the affinity for IMPα ∼300 fold. Consistently, substitution of ^Prp20NLS^K^20^ to Thr severely disrupts Prp20 interaction with IMPα to approximately 20% of that of the wild-type protein [16]. That the larger arginine side chain is less energetically favourable in the P2 position has also been demonstrated through a K128R substitution in the SV40 TAg cNLS, which resulted in a ∼3 kcal/mol decrease in binding free energy. This high conservation of P2 lysine is rationalized through the specific and extensive hydrogen-bonding interactions with IMPα; the terminal nitrogen atom of the lysine side chain coordinates with the main chain carbonyl group of Gly^150^, with the hydroxyl in the side chain of Thr^155^, and with the negatively charged side chain of Asp^192^. These precise interactions were also observed in our structure. Furthermore, ^Prp20NLS^Met^21^ occupies the P3 position in our structure, and interacts with the side chains of Trp^184^ and Asn^188^. This is consistent with nuclear import assays in yeast, which showed no defects in Prp20 import when Met^21^ was substituted to a Thr residue [16]. The P4-binding site exhibits a slight preference for arginine, because it is able to make the most favourable interactions with ARM repeats 1 and 2; however a greater tolerance within this binding pocket has been noted than for the P2 position, and consistently, the energy contribution from this pocket is ∼1/4 of the contribution of the P2 residue.

The minor site P1′ and P2′ positions contain the ‘KR’ motif in nearly all IMPα:NLS structures solved to date, with the replacement to non-KR residues commonly resulting in cytoplasmic localisation of the protein. The P1′-binding pocket is generally defined by residues Thr^328^ and Asp^361^, and whilst a lysine is preferred over arginine in this binding cavity (which was observed in our structure), because an arginine side chain at this position is too long to make optimal interactions with the IMPα side chains, arginine can still be accommodated, e.g. in the case of CBP80 [6]. The P2′-binding pocket is defined by residues Ser^360^ and Glu^396^ within ARM repeats 7 and 8. Conversely to P1′, whilst a lysine can be accommodated at this position, an arginine side chain is able to make more favourable contacts to the IMPα minor binding site, and the Prp20 NLS therefore contains the most favoured arrangement KR motif at P1′ and P2′ positions, forming both specific side chain salt bridges, and main chain hydrogen-bonding interactions. Mutations of these residues have shown a weaker interaction to IMPα and defects in nuclear localisation in yeast cells [16].

Overall, our structure defines the binding mechanism of the bipartite Prp20 NLS with the nuclear import receptor IMPα. This interaction has not been described previously, and importantly, provides new structural information relating the mechanism of IMPα NLS recognition. The linker region, separating the positively charged clusters within the bipartite NLS of Prp20, whilst longer than many bipartite NLS linkers, does not perturb the ability of these clusters to interact with the major and minor sites of IMPα in a manner characteristic of other bipartite NLSs. Whilst insertion of a residue between the classical P0 and P1 disrupted the classical binding observed at P0, this was compensated by additional binding observed in the close vicinity, and highlights both the flexibility of NLS recognition contained within IMPα, and difficulty in precisely predicting NLSs.
